# Enhanced repair of DNA interstrand crosslinking in ovarian cancer cells from patients following treatment with platinum-based chemotherapy

**DOI:** 10.1038/sj.bjc.6603973

**Published:** 2007-09-11

**Authors:** P Wynne, C Newton, J A Ledermann, A Olaitan, T A Mould, J A Hartley

**Affiliations:** 1 Cancer Research UK Drug-DNA Interactions Research Group, UCL, London, UK; 2 Department of Oncology, Royal Free and University College Medical School, UCL, London, UK; 3 UCLH Gynaecological Cancer Centre, London, UK

**Keywords:** ovarian cancer, drug resistance, DNA crosslinking, DNA repair, platinum chemotherapy

## Abstract

Despite high tumour response rates to platinum-based chemotherapy in ovarian cancer survival is poor due to the emergence of drug resistance. Mechanistic studies in clinical material have been hampered by the unavailability of sensitive methods to detect the critical drug-induced effects in individual cells. A modification of the single cell gel electrophoresis (comet) assay allows the sensitive detection of DNA interstrand crosslinking in both tumour and normal cells derived directly from clinical material. Tumour cells isolated from 50 ovarian cancer patients were treated *ex vivo* with 100 *μ*M cisplatin for 1 h and crosslink formation and repair (unhooking) measured. No significant difference in the peak level of crosslinking in tumour cells was observed between patients who were either newly diagnosed or previously treated with platinum-based therapy, or between tumour and mesothelial cells from an individual patient. This indicates no difference in cellular mechanisms such as drug transport or detoxification. In contrast, the percentage repair (unhooking) of DNA interstrand crosslinks was much greater in the group of treated patients. At 24 h in the 36 newly diagnosed patient tumour samples, only one gave >50% repair and 23 gave <10% repair; however, 19 out of 22 treated patient samples gave >10% repair and 14 showed >50% repair. The estimated median difference (newly diagnosed minus treated) was −52 (95% CI −67 to −28), and the *P*-value from a Mann–Whitney test was <0.001. In eight patients, it was possible to obtain tumour samples prior to any chemotherapy, and also on relapse or at interval debulking surgery following platinum-based chemotherapy. In these patients, the mean % repair prior to therapy was 2.85 rising to 71.23 following treatment. These data demonstrate increased repair of DNA interstrand crosslinks in ovarian tumour cells following platinum therapy which may contribute to clinical acquired resistance.

The lifetime risk of a woman developing ovarian cancer is 1 in 70 and around two thirds of these patients present with advanced disease (Ozols *et al*, 2001). The standard first line treatment for ovarian cancer is cytoreductive surgery followed by carboplatin alone or more commonly in combination with paclitaxel (du Bois *et al*, 2005). This treatment results in a complete response in the majority of women; however, most responding patients eventually relapse with disease that becomes resistant not only to platinum compounds, but also to a wide range of other chemotherapeutic agents (Salzberg *et al*, 2005). The prognosis for women with relapsed ovarian cancer remains poor with a 5-year survival of 25% (Colombo *et al*, 2006). A greater understanding of the mechanisms underlying drug resistance could lead to measures to overcome it and improve survival.

Resistance to chemotherapeutic agents such as carboplatin can be intrinsic or acquired (Perez, 1998). Intrinsic resistance is present at the time of diagnosis, and the patient fails to respond to first line chemotherapy. Studies in ovarian cancer cell lines have shown that acquired resistance to platinum drugs can be multifactorial, consisting of mechanisms which include altered drug transporter proteins (Kelland, 2000), increased drug inactivation, for example, by binding of drugs to glutathione (Kelland, 2000), evasion of apoptosis by mutation of genes such as p53 (Manic *et al*, 2003) and enhanced ability to repair DNA damage such as by upregulation of ERCC1 (Ferry *et al*, 2000). It is still unclear, however, which of these contribute most to acquired drug resistance in the clinical setting. Studies in clinical material have been hampered by the unavailability of sensitive methods to detect the critical drug-induced effects in individual cells.

The cytotoxicity of carboplatin and cisplatin results from the formation of platinum-DNA adducts which include monoadducts, intrastrand crosslinks, interstrand crosslinks (ICLs) and DNA-protein crosslinks (Zwelling *et al*, 1979; Comess and Lippard, 1993). Intrastrand crosslinks constitute the majority (>80%) of lesions formed on cellular DNA and these distorting lesions are repaired by nucleotide excision repair (Fichtinger-Schepman *et al*, 1995). ICLs, which link the two complementary strands of DNA together, comprise less than 5% of the total lesions on DNA but are highly cytotoxic and difficult to repair (McHugh *et al*, 2001).

We have previously demonstrated that a modification of the single cell gel electrophoresis (comet) assay (Spanswick *et al*, 1999) can be used successfully in the clinical setting to detect and quantify the levels of ICLs in patient lymphocytes and tumour cells at pharmacologically relevant doses of bifunctional alkylating agents (Hartley *et al*, 1999; Webley *et al*, 2001; Spanswick *et al*, 2002; Corrie *et al*, 2005). The method has also been used to measure cisplatin-induced ICLs *in vitro* (De Silva *et al*, 2002). In this study, we have used the method to compare the formation and repair (unhooking) of ICL's following *ex vivo* exposure to cisplatin in tumour cells and normal cells isolated from ovarian cancer patients who were either newly diagnosed, or had been previously treated with platinum-based chemotherapy.

## MATERIALS AND METHODS

### Patient population

Ethics approval was gained from the Joint UCL/UCLH Committee on the Ethics of Human Research. Ovarian cancer patients receiving treatment between February 2001 and February 2006 were recruited to take part in this study. Solid tumour tissue or ascitic fluid was obtained from 50 ovarian cancer patients aged between 45 and 91 years. Samples were obtained at diagnosis, interval debulking surgery (IDS) or at relapse. In some cases, paired samples were obtained at diagnosis and IDS, or at relapse.

### Preparation of tumour and non-tumour cells from clinical material

Ascitic fluid was aliquoted into plastic 50 ml conical tubes and spun at 200 × *g* for 5 min. Cell pellets were resuspended in Dulbecco's modification of Eagle's medium (DMEM) containing 10% fetal calf serum (FCS) and 2 mM glutamine, and seeded into large tissue culture flasks. All cells were maintained in a humidified atmosphere with 5% CO_2_ at 37°C. After 1 h, the entire volume of tissue culture medium in each flask, containing unattached cells was transferred into a fresh large tissue culture flask, and DMEM (with FCS and glutamine) was replaced in the original flasks. Non-tumour cells generally attached to the plastic surface within the first hour, whereas tumour cells required a longer period of incubation. Tumour cells also required a longer period of time to detach in response to trypsin, compared to the non-tumour, mesothelial cells. Further purification of the tumour samples was achieved by trypsinisation until the contaminant mesothelial cells were seen to detach, while the tumour cells remained *in situ*.

In sterile conditions, primary tumour was finely dissected and flushed with DMEM containing 10% FCS and 2 mM glutamine, to produce a single cell suspension, which was seeded into large tissue culture flasks.

### Immunocytochemistry

Antibodies to CA125 (Novocastra, Newcastle, UK) and AUA1 (Skybio, Bedfordshire, UK) which stain ovarian tumour cells and not mesothelial cells, and to Calretinin (Zymed, Cambridge, UK) and CK5 which stain mesothelial cells but not ovarian cancer cells, were used to differentiate the two cell types in cytospin preparations using standard immunocytochemical techniques. Analysis was performed on the same cell population that was treated with cisplatin.

### Treatment of tumour and non-tumour cells *ex vivo* with cisplatin

Primary cultures of tumour and mesothelial cells were trypsinised and seeded at a concentration of 5 × 10^4^ cells per ml into 6-well plates. Cells were left to attach overnight. Half of the samples were non-drug-treated controls, the other half were drug treated with 100 *μ*M cisplatin (David Bull Laboratories, Australia), diluted in DMEM, for 1 h at 37°C in a humidified atmosphere with 5% CO_2_. The cisplatin was removed and fresh DMEM with 10% FCS and 2 mM glutamine was added to the samples. Immediately after drug treatment, a drug treated and a non-drug-treated control sample were trypsinised, centrifuged at 200 **g** for 5 min, then resuspended in FCS with 10% DMSO. Samples were then frozen in a polystyrene box within a −80°C freezer. This procedure was repeated 4.5, 9, 24 and 48 h after drug exposure.

Single cell suspensions were prepared at a cell density of 5 × 10^4^ cells per ml. Cells were treated with 100 *μ*M cisplatin in DMEM at 37°C, 5% CO_2_. After exposure, cell samples were centrifuged at 200 **g** for 5 min, and then resuspended with DMEM with 10% FCS and 2 mM glutamine. Immediately after drug treatment, a drug treated and a non-drug-treated control samples were trypsinised, centrifuged at 200 **g** for 5 min, and then resuspended in FCS with 10% DMSO. Samples were then frozen in a polystyrene box within a −80°C freezer. This procedure was repeated 4.5, 9, 24 and 48 h after drug exposure.

### Measurement of DNA interstrand crosslinking using the single cell gel electrophoresis (comet) assay

The details of the modified single cell gel electrophoresis (comet) assay to measure DNA ICLs are described in detail elsewhere (Hartley *et al*, 1999; Spanswick *et al*, 1999). All procedures performed on the single cell suspension sample were carried out on ice and in subdued lighting. All chemicals used were obtained from Sigma Chemical Co. (Poole, UK) unless otherwise stated. Immediately before analysis, cells were irradiated (12.5 Gy, 2.35 Gy min^−1^) to deliver a fixed number of random DNA strand breaks. After embedding cells in 1% agarose on a precoated microscope slide, the cells were lysed for 1 h in lysis buffer (100 mM disodium EDTA, 2.5 M NaCl, 10 mM Tris-HCl pH 10.5) containing 1% Triton X-100 added immediately before analysis, and then washed for 1 h in distilled water, changed every 15 min. Slides were then incubated in alkali buffer (50 mM NaOH, 1 mM disodium EDTA, pH 12.5) for 45 min followed by electrophoresis in the same buffer for 25 min at 18 V (0.6 V cm^−1^), 250 mA. The slides were finally rinsed in neutralising buffer (0.5 M Tris-HCl, pH 7.5) and then in saline.

After drying, the slides were stained with propidium iodide (2.5 *μ*g ml^−1^) for 30 min and then rinsed in distilled water. Images were visualised using a NIKON inverted microscope with a high-pressure mercury light source, 510–560 nm excitation filter and 590 nm barrier filter at × 20 magnification. Images were captured using an online CCD camera and analysed using Komet Analysis software (Kinetic Imaging, Liverpool, UK). For each duplicate slide, 25 cells were analysed. The tail moment for each image was calculated using the Komet Analysis software as the product of the percentage DNA in the comet tail and the distance between the means of the head and tail distributions, based on the definition of Olive *et al* (1990). Crosslinking was expressed as the percentage decrease in tail moment compared to irradiated controls calculated by the formula: 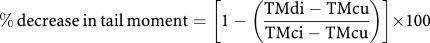 where Tmdi is the tail moment of drug-treated irradiated sample, TMcu the tail moment of untreated, unirradiated control and TMci the tail moment of untreated, irradiated control.

### Statistical analysis

Statistical analyses were performed in Minitab version 13.32. Probability plots were observed to determine whether the three variables (percentage decrease in tail moment, the paired difference in tail moment decrease between tumour and mesothelial cells, and percentage repair at 24 h) were normally distributed. If they were, unpaired or paired *t*-tests were performed, and the mean difference with 95% CI obtained. If the distributions were not normal, the median was used as the measure of central tendency and the Mann–Whitney non-parametric test used to examine differences between groups. Minitab also provides an estimate of the median difference between newly diagnosed and treated patients, with 95% CI.

## RESULTS

### Measurement of DNA interstrand crosslinking in ovarian tumour cells treated *ex vivo* with cisplatin using the single cell gel electrophoresis (comet) assay

Samples were obtained from 36 patients prior to any platinum-based chemotherapy (Table 1A) and from 22 patients following platinum-based chemotherapy (Table 1B). In eight cases (patients 3, 17, 18, 27, 34, 39, 44 and 47) paired samples were obtained at diagnosis, and at relapse or IDS following platinum-based chemotherapy. Either primary tumour cell cultures from drained ascitic fluid or single cell suspensions from ovarian tumours from surgery were obtained. Immunohistochemistry was used to determine the purity of the tumour cell population. In all cases, the cell sample contained >80% tumour cells and in the majority of cases it was >90%.

**Figure 1:**
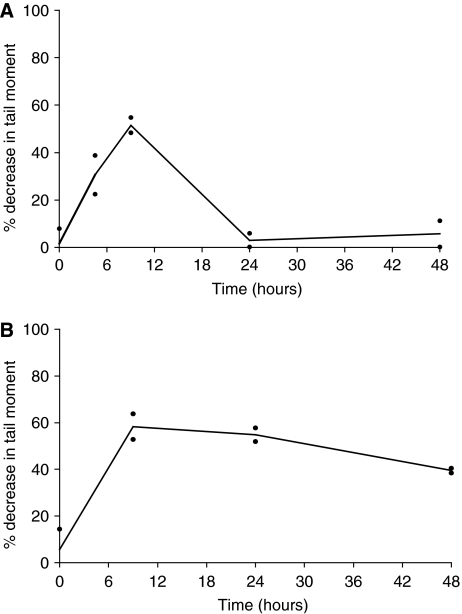
Time course of DNA interstrand crosslink formation and repair in human ovarian cancer cells from patient 1 (**A**) and patient 8 (**B**) as determined by the single cell gel electrophoresis (comet) assay. Cells were treated *ex vivo* with cisplatin for 1 h at 100 *μ*M. The data points are the values from two independent experiments and the lines are plotted through the mean.

Cells were treated with cisplatin for 1 h at 100 *μ*M. This dose was determined from pilot experiments in human ovarian cancer cell lines to give an optimal level of DNA ICLs as determined by the single cell gel electrophoresis (comet) assay. Following 1 h treatment cells were re-suspended in drug-free medium and samples taken for measurement of DNA crosslinking at 9 h (the peak of crosslinking with cisplatin), 24 and 48 h. DNA crosslinking was expressed as the % decrease in tail moment compared to control non-drug treated cells as previously described (Hartley *et al*, 1999; Spanswick *et al*, 1999). Crosslink response curves for patient samples 1 and 8 are shown in Figure 1. In the tumour cells from patient 1 (Figure 1A) around 55% decrease in tail moment is observed at the peak of crosslinking (9 h). By 24 h, the majority (>95%) of the crosslinks have been repaired or ‘unhooked’ from the DNA. In contrast, in the cells from patient 8 (Figure 1B) although the level of crosslinks at the peak is similar, very little unhooking is observed at 24 h (<10%) and the majority of crosslinks persist at 48 h.

### Peak level of cisplatin-induced DNA interstrand crosslinks in patient tumour and mesothelial cells

**Figure 2:**
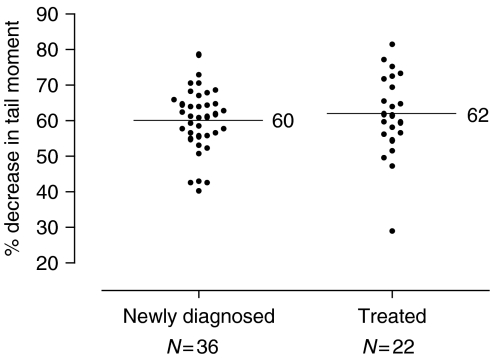
Level of DNA interstrand crosslinking at 9 h following treatment with 100 *μ*M cisplatin in tumour cells from 50 patients as determined by the comet assay. Scatter plot of the percentage decrease in tail moment in newly diagnosed patients, and those previously treated with platinum-based chemotherapy. The horizontal lines indicate the mean value in each group.

The peak (9 h) level of DNA interstrand crosslinking was determined in all patient tumour samples, following treatment with 100 *μ*M cisplatin. The data are presented in Figure 2 with the newly diagnosed patients and those treated with platinum-based chemotherapy shown separately. A high level of crosslinking was observed in all the samples tested with the % decrease in tail moment ranging from 30 to 81%. The mean level of crosslinking in all samples was 60.54. The percentage decrease in tail moment was normally distributed. The mean difference between the two groups (newly diagnosed minus treated) is −2, with 95% CI −7 to 4. The *P*-value from an unpaired *t*-test was 0.49, indicating no evidence of a real difference.

**Figure 3:**
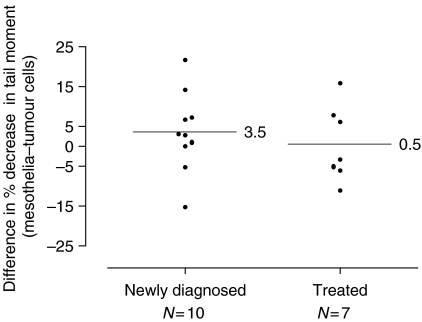
DNA interstrand crosslinking at 9 h following treatment with 100 *μ*M cisplatin in tumour cells and mesothelial cells isolated from the same patient. Scatter plot shows the difference in the percentage decrease in tail moment in newly diagnosed and previously treated patients. The horizontal lines indicate the mean value in each group.

In many of the samples derived from ascitic fluid, it was possible to isolate mesothelial cells to act as a non-tumour direct comparison within the same patient. Data for the matched samples are shown in Figure 3. The paired difference between the percentage decrease in tail moment (mesothelial minus tumour cells for each patient) was normally distributed. In the 10 newly diagnosed cases, the mean difference between tumour and control cells was 3.5 (95% CI −3.7 to 10.7), with a *P*-value from a paired *t*-test of 0.30. In the seven treated cases, the mean difference was 0.5 (95% CI −8.3 to 9.3), *P*-value of 0.90. Therefore, in each group, there was no evidence of a difference between tumour and mesothelial cells. These data demonstrate that tumour cells and mesothelial cells do not differ significantly in their uptake or cellular metabolism of cisplatin thereby allowing similar levels of DNA damage to occur.

### Repair of cisplatin-induced crosslinks in patient tumour cells

**Figure 4:**
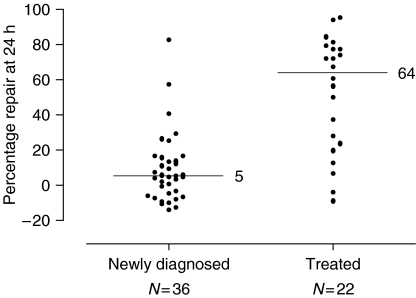
Repair (unhooking) of cisplatin-induced DNA interstrand crosslinking in the same tumour samples as Figure 2. Data are expressed as the % repair at 24 h compared to the peak level at 9 h. A negative % repair indicates that the level of crosslinking was higher at the 24 h time point than at the 9 h point. Scatter plot of the percentage repair at 24 h in newly diagnosed and previously treated patients. The distributions are skewed, so the horizontal lines indicate the median value in each group.

The ability of the tumour cells to repair the DNA ICLs produced by cisplatin was determined from the crosslink response curves produced for each patient sample. The level of crosslinking was compared at 9 and 24 h and the % repair at 24 h calculated. These data are shown in Figure 4. A highly heterogeneous response was observed between the different patient samples ranging from no repair to almost 100% repair at 24 h. In some samples, the level of crosslinking was even slightly higher at 24 h than at 9 h resulting in a ‘negative’ % repair value. Strikingly, the response in the samples from newly diagnosed patients was generally very different to that in the samples from platinum-treated patients. In the 36 newly diagnosed patients, only one gave a level of repair above 50% and 23 gave <10% repair. In contrast, 19 out of 22 previously treated patients gave >10% repair and 14 showed >50% repair. The mean % repair was 8.75 in the newly diagnosed patients compared to that of 52.4 in the treated patients. Percentage repair at 24 h was not normally distributed. The estimated median difference (newly diagnosed minus treated) is −52 (95% CI −67 to −28), and the *P*-value from a Mann–Whitney test was <0.001. These results show that the percentage repair was much greater in the group of treated patients.

In the mesothelial samples, the repair response was more homogeneous than in the matched tumour samples. The mean % repair in the 17 mesothelial samples was 17.84±20.77 (18.82±24.41 in the 10 samples from newly diagnosed patients and 16.43±15.9 in the seven samples from treated patients).

### Repair of cisplatin-induced crosslinking in tumour cells from the same patient before and after platinum-based chemotherapy

**Figure 5:**
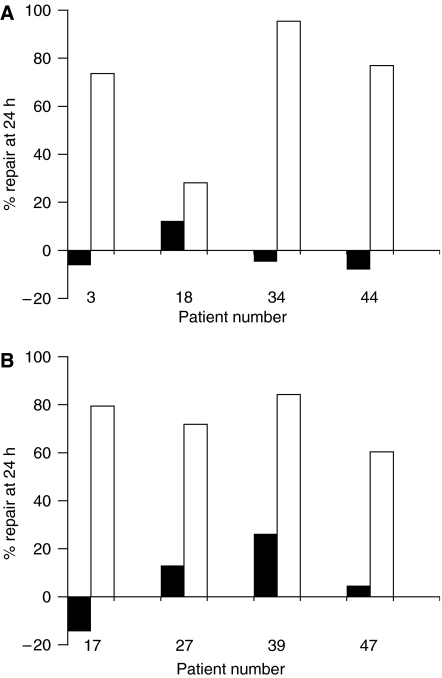
Repair of cisplatin-induced DNA interstrand crosslinking in tumour from eight patients where samples were taken both at initial diagnosis and following platinum-based chemotherapy. In (**A**) the second samples were taken at interval debulking surgery and in (**B**) the samples were taken at relapse following a treatment-free interval <6 months.

In eight patients, it was possible to obtain tumour samples prior to any chemotherapy, and also on relapse following platinum-based chemotherapy. The % repair values for these patients are shown in Figure 5. In four patients, the second sample was taken at IDS (Figure 5A) and in the other four, the second sample was taken at relapse following a treatment-free interval <6 months (Figure 5B). These two clinically distinct groups showed similar changes. In five out of the eight patients prior to chemotherapy the tumour cells did not show any repair of crosslinks at 24 h, and <30% repair at 24 h was observed in the other three. In contrast, the tumour cells following chemotherapy show extensive unhooking of crosslinks in each case with % repair ranging from 28.1 to 95.4, with seven of the eight samples showing >60% repair. In these eight patients, the mean % repair prior to therapy was 2.85±13.59 rising to 71.23±20.12 following treatment.

## DISCUSSION

The data presented here clearly demonstrate that, in tumour cells isolated from 50 ovarian cancer patients, the peak level of DNA interstrand crosslinking produced by the chemotherapeutic drug cisplatin is very similar (mean 60.54±9.98), as determined by the single cell gel electrophoresis (comet) assay. This is irrespective of whether the tumour sample was from a newly diagnosed patient, or one who had been treated with platinum-based chemotherapy. This would indicate that any molecular mechanism of drug resistance that has been evoked following chemotherapy does not involve an ‘upstream’ mechanism (e.g., altered drug transport, increased detoxification) which would prevent the drug from reaching its cellular target, DNA. Similarly, tumour cells and mesothelial cells from the same patient do not differ significantly in their uptake or cellular metabolism of cisplatin thereby allowing similar levels of DNA damage to occur.

The single cell gel electrophoresis (comet) assay allows DNA interstrand crosslinking to be measured in clinical samples at pharmacologically relevant doses of crosslinking drug. This can be used to measure crosslinking in lymphocytes or solid tumour material where samples are taken following treatment of patients with drugs such as ifosfamide (Hartley *et al*, 1999), treosulfan (Corrie *et al*, 2005) or antibody directed enzyme pro-drug therapy (Webley *et al*, 2001). Alternatively, it can be used to measure crosslink formation and repair in cells isolated from patients and treated *ex vivo* with drug as in the present study, or as previously demonstrated in myeloma plasma cells treated with melphalan (Spanswick *et al*, 2002). In the latter study, myeloma cells from chemotherapy naïve patients were all incapable of repairing melphalan-induced crosslinks at 24 h after the peak of formation. Cells from melphalan resistant patients all showed significant repair ranging from 42 to 100% repair at 24 h. In the current study, the repair of crosslinking was more heterogeneous in both the newly diagnosed and treated patient populations but the overall trend to increased repair of interstrand crosslinking was clearly evident.

It should be noted that repair as measured by the comet assay is really the ‘unhooking’ of one arm of the crosslink to release the covalent linkage of the two strands of the double helix. This is the first step in the complex molecular mechanism of repair of DNA ICLs (McHugh *et al*, 2001) and the comet assay cannot determine if the repair process has gone to completion and correctly restored the integrity of both strands of the DNA. Mammalian cells defective in the unhooking step of cisplatin interstrand crosslink repair, as measured using the comet assay, include cells bearing mutations in nucleotide excision repair (e.g., XPB, XPD, XPG, ERCC1 and XPF) and homologous recombination (e.g., XRCC2 and XRCC3) (De Silva *et al*, 2002). Cells defective in ERCC1 are highly sensitive to cisplatin and several groups have investigated the influence of ERCC1 on resistance to platinum chemotherapy (Ferry *et al*, 2000; Reed, 2005) and suggest that ERCC1 is a good marker for cellular or clinical resistance to these drugs. In ERCC1 mutant cells, however, the high cisplatin sensitivity observed compared to other mutant cells which are equally defective in the unhooking step of interstrand crosslink repair is most likely due to a defect other than in excision repair (De Silva *et al*, 2002).

It has previously been demonstrated that the repair of DNA ICLs produced by cisplatin, measured by the technique of alkaline filter elution, was reduced in human lymphocytes from normal volunteers aged around 70 compared to those from volunteers aged around 20 (Rudd *et al*, 1995). In the current study, the age range of patients was from 45 to 91 and there was no correlation between age and extent of repair in the newly diagnosed patient tumour samples.

Platinum compounds are the most active agents in ovarian cancer treatment and the decision to retreat recurrent disease with platinum is based on clinical observations that have shown the likelihood of response is dependent on the platinum-free interval (Blackledge *et al*, 1989; Markman *et al*, 1991). The study of *ex vivo* treatment of tumour samples from women with newly diagnosed and relapsed ovarian cancer has identified biochemical changes in the formation and repair of cisplatin-induced DNA crosslinks that provide new information on some of the mechanisms associated with resistance to platinum in patients with ovarian cancer. Firstly, the ability of cisplatin to form DNA crosslinks is similar in normal (mesothelial) and tumour tissue and similar levels of crosslinking were seen in patients whose tumours were exposed to *in vitro* cisplatin after a ‘platinum-free interval’ of less than or greater than 6 months. Secondly, in comparison to the platinum naïve group, there were marked differences in the repair of platinum-induced crosslinks in tumour cells removed at IDS or after relapse at a less than or greater than 6 months platinum-free period. In the previously treated group, 86% showed greater than 10% repair compared with 36% in chemonaïve patients.

A Cox regression was used to examine the association between progression-free survival and percentage DNA repair in the newly diagnosed patient samples. There was no evidence of an association (the hazard ratio for an increase of 1 percentage point was 0.99, 95% CI 0.97–1.02, *P*-value=0.56). No relationship was therefore evident between repair of ICLs and inherent sensitivity. In the case of treated patients, the number in each category (IDS, platinum-free interval (PFI) >6 months, PFI <6 months) was too small to perform the equivalent analysis.

The paired samples (Figure 5) allow further conclusions to be drawn. In all four paired samples taken pre-treatment and then at IDS there was an increased ability to repair platinum-induced crosslinks after 3–4 cycles of platinum-based chemotherapy. This suggests that significant changes in the tumour have either developed, or become evident through selection after as few as three cycles of chemotherapy. In three of these cases, the outcome after chemotherapy and surgery was a complete response, but relapse occurred between 4 and 12 months in all four cases. As previously stated, the unhooking of DNA crosslinks is only one of a number of events leading to repair and contributing to clinical resistance. Even in a larger group of patients, it is unclear whether this early change in the tumour metabolism has clinically meaningful information. Similarly, within the sub-group of clinical ‘platinum-sensitivity’ or ‘-resistance’, it is difficult to draw conclusions about a relationship of repair to progression-free survival on further treatment as the number of patients per group is small and the treatment given at relapse varied. Furthermore, the definition of platinum-sensitivity is a clinical one and represents an empirically defined grouping of patients, based on an observed probability of response to platinum re-challenge. However, there is a consistent pattern within the paired samples.

For the four patients with paired samples at relapse/progression, a significant increase in repair was also seen compared to their pre-treatment sample. Three patients were considered too unwell for further treatment at this point but one (patient 39), treated with cisplatin and etoposide had a partial response lasting more than 4 months. Whilst this study does not assist the clinical decision process about the choice of therapy for first or subsequent line therapy, it clearly shows that changes in the tumour metabolism of cisplatin-induced crosslinks evolve quickly after platinum-based therapy and that the mechanisms of clinical resistance are likely to involve the repair and processing of DNA ICLs. The early appearance of these differences merits further investigation in a larger number of patients treated with platinum-based therapy to determine any relationship between this enhanced DNA repair and clinical outcome.

## Figures and Tables

**Table 1A:** Newly diagnosed patient characteristics

**Patient number**	**Age**	**Treatment post sample**	**FIGO stage**	**Progression-free survival (months)**	**Sample type**
2	56	Carboplatin/paclitaxel	3c	>55	Ascites
3	48	Carboplatin/paclitaxel	3c	15	Ascites
5	50	Carboplatin/paclitaxel	4	9	Ascites
6	57	Carboplatin/paclitaxel	4	20	Ascites
8	60	Carboplatin	3c	0	Ascites
9	78	Carboplatin	3c	12	Ascites
12	58	Carboplatin/paclitaxel	3c	17	Ascites
17	64	Carboplatin	3c	10	Ascites
18	63	Carboplatin/paclitaxel	4	12	Ascites
19	68	Carboplatin/paclitaxel	2c	>13	Solid tumour
20	91	None	Not known	NA	Ascites
21	65	Carboplatin/paclitaxel	3c	>4	Solid tumour
23	74	Carboplatin/paclitaxel	3c	10	Ascites
24	63	Carboplatin/paclitaxel	3c	7	Ascites
25	50	Carboplatin/paclitaxel	2c	>4	Solid tumour
26	63	Carboplatin/paclitaxel	3c	>4	Solid tumour
27	54	Carboplatin/paclitaxel	3c	0	Ascites
28	45	Carboplatin/paclitaxel	3a	>8	Solid tumour
29	77	Carboplatin	2b	>6	Solid tumour
30	62	Carboplatin	1c	>6	Ascites
32	62	Carboplatin	2b	>7	Solid tumour
33	73	Carboplatin/paclitaxel	3c	>7	Ascites
34	70	Carboplatin/paclitaxel	3c	9	Ascites
36	61	Carboplatin	1a	>4	Solid tumour
37	54	None (too unwell)	3c	0	Ascites
38	64	Carboplatin/paclitaxel	3	3	Ascites
39	58	Carboplatin/paclitaxel	4	0	Ascites
40	63	Carboplatin/paclitaxel	3c	5	Ascites
42	87	Carboplatin	3c	>6	Solid tumour
44	80	Carboplatin	3c	6	Ascites
45	76	Carboplatin	3c	>4	Ascites
46	78	None (too unwell)	3c	NA	Ascites
47	77	Carboplatin (intraperitoneal)	3c	0	Ascites
48	66	Carboplatin/paclitaxel	4	>3	Ascites
49	56	No data	3c	No follow up	Ascites
50	66	Carboplatin/paclitaxel	3c	>4	Ascites

Abbreviation: NA=not applicable.

**Table 1B:** Treated patient characteristics (IDS and relapse)

**Patient number**	**Age**	**Treatment post sample**	**FIGO stage**	**PFI months**	**Clinical Category**	**PFS months**	**Sample type**
3	48	Carboplatin/paclitaxel	3c	NA	IDS	15	Solid tumour
15	51	Carboplatin/paclitaxel	4	NA	IDS	>36	Solid tumour
18	63	Carboplatin/paclitaxel	4	NA	IDS	11	Solid tumour
34	70	Carboplatin/paclitaxel	3c	NA	IDS	9	Ascites
35	53	Etoposide	4	NA	IDS	11	Solid tumour
43	69	Cisplatin/etoposide	3c	NA	IDS	0	Ascites
44	80	Carboplatin	3c	NA	IDS	>6	Ascites
1	53	Carboplatin	4	24	>6months^a^	5	Ascites
4	59	Carboplatin	4	27	>6months	9	Ascites
14	57	Topotecan	3c	8	>6months	0	Ascites
7	68	Topotecan	3c	6	<6months^a^	11	Ascites
10	71	Liposomal doxorubicin	3c	4	<6months	4	Ascites
11	56	Liposomal doxorubicin	3c	2	<6months	0	Solid tumour
13	75	Carboplatin/gemcitabine	4	0	<6months	0	Ascites
16	80	None^b^	3c	0	<6months	0	Ascites
17	66	None	3c	3	<6months	0	Ascites
22	69	None^b^	4	0	<6months	0	Ascites
27	54	None^b^	3c	0	<6months	0	Ascites
31	49	Carboplatin/paclitaxel	3c	4	<6months	0	Ascites
39	58	Cisplatin/etoposide	4	0	<6months	>6	Ascites
41	63	None^b^	4	4	<6months	0	Ascites
47	77	None^b^	3c	0	<6months	NA	Ascites

Abbreviations: IDS=interval debulking surgery; NA=not applicable; PFI=platinum-free interval; PFS=progression-free survival.

a Clinical category of relapse determined by the PFI is used to decide about likelihood of a response to further platinum-based chemotherapy.

b Patients relapsed on carboplatin chemotherapy.
